# Transrectal ultrasonographic parameters combined with total serum exosomes for the assessment of bone metastases in prostate cancer

**DOI:** 10.1515/biol-2025-1358

**Published:** 2026-09-04

**Authors:** Lingxu Cui, Hua Hong, Guohua Wu, Qian Liu

**Affiliations:** Department of Functional Examination, University Hospital of Inner Mongolia for Nationalities, Tongliao 02800, Inner Mongolia, China; Department of Ultrasound, Inner Mongolia People’s Hospital, Hohhot 010017, Inner Mongolia, China; Inner Mongolia Medical University, Hohhot 010107, Inner Mongolia, China

**Keywords:** prostate cancer, bone metastasis, transrectal ultrasonography, contrast parameters, total serum exosomes

## Abstract

This study investigated transrectal ultrasound parameters and total serum exosome levels in prostate cancer (PCa) patients with bone metastasis (BM), and evaluated their combined value in assessing metastatic status. 40 PCa patients were categorized into non-bone metastasis (NBM, 21 cases) and BM (19 cases) groups based on single photon emission computed tomography/computed tomography or bone scans. Serum exosomes were extracted and analyzed via nanoparticle tracking analysis, while transrectal ultrasound and ultrasound-guided biopsies were performed. The study compared exosome levels and ultrasound parameters between groups, and assessed their diagnostic value for BM using receiver operating characteristic (ROC) curves, and analyzed correlations between exosomes and ultrasound findings. PCa patients with BM showed significantly higher serum exosome concentrations and ultrasound parameters (peak intensity, area under the curve (AUC), and Grad) than NBM patients (p < 0.001). ROC analysis revealed optimal cutoff values for discriminating BM: 19.33 dB for PI, 1,156.63 dB s for AUC, 2.02 dB/s for Grad, and 3.34 × E^10^ particles/mL for exosomes. The combined test (AUC = 0.97) outperformed individual parameters (AUCs 0.83–0.89), demonstrating superior diagnostic performance in identifying PCa BM. In conclusion, this exploratory preliminary study indicates that exosomes may have potential application value in the assessment of PCa BM.

## 1 Introduction

As prostate cancer (PCa) has an insidious onset and atypical clinical manifestations in the early stage. Patients are often in the middle or late stage when they present with symptoms to the doctor, thus missing the best period of treatment. The metastasis rate among new PCa patients in China is as high as two-thirds, and studies have found that when PCa metastasis occurs, the probability of bone metastasis (BM) is the greatest, which can reach more than 90 % [1]. Therefore, it is important to clarify whether BM develops in PCa as early as possible in order to formulate individualized treatment plans and improve patients’ prognosis. At this stage, single photon emission computed tomography/computed tomography (SPECT/CT) bone imaging is mostly used to diagnose BM of PCa, but its specificity is low and its cost is higher. Contrast-enhanced ultrasound (CEUS) can objectively reflect the perfusion status of tissues and show the neovascularization associated with the development of PCa [2], which can help to identify PCas with different degrees of malignancy. Exosomes are a kind of extracellular vesicles spontaneously secreted by cells, which can carry lipids, proteins, nucleic acids and other substances, and are involved in the development of PCa, angiogenesis, and other processes [3]. In this cross-sectional study, we investigated transrectal ultrasonographic parameters and total serum exosome concentrations in PCa patients with BM, and explored their combined utility in the evaluation of metastatic involvement.

## 2 Materials and methods

### 2.1 Subjects of the study

A total of 40 PCa patients who were treated at the Department of Urology, the Inner Mongolia People’s Hospital between January 2022 and December 2022 were selected for the study. The patients’ ages ranged from 59 to 85 years (71.75 ± 7.38 years). Inclusion criteria: (1) patients with PCa diagnosed for the first time by puncture biopsy or postoperative pathology; (2) no history of surgery or endocrine treatment of the prostate; (3) The imaging data of transrectal contrast-enhanced ultrasound, SPECT/CT or MRI were complete and the images were clear without artifacts. Exclusion criteria: (1) patients with concurrent other malignant tumor diseases or congenital bone disease in whom the cause of BM could not be identified; (2) patients with concurrent severe heart and liver diseases.

**Informed consent:** Informed consent was obtained from all individuals included in this study, or their legal guardians or wards.

**Ethical Approval:** The research related to human use has been complied with all the relevant national regulations, institutional policies and in accordance with the tenets of the Helsinki Declaration, and has been approved by the Ethics Committee of University Hospital of Inner Mongolia for Nationalities.

### 2.2 Criteria to be fulfilled for the diagnosis of BM in PCa

BM was diagnosed based on the following three criteria, all of which had to be met simultaneously: (1) a clearly documented history of PCa, with exclusion of patients suffering from malignant tumors of other organs and patients with a history of fracture or bone trauma; (2) review by the nuclear medicine department showed that pre-existing foci had not disappeared but rather had progressed or increased in number; and (3) osteogenic or osteolytic bone destruction was seen by SPECT/CT. The images were independently reviewed by two chief nuclear medicine physicians who were blinded to the patients’ clinical and ultrasound contrast imaging results.

### 2.3 Ultrasonography

GE LOGIQE9 ultrasound diagnostic instrument was used. The patients were placed in the left lateral position, and a routine transrectal ultrasound examination was performed to observe the morphology of the prostate gland, internal echoes, and blood flow distribution, etc. Abnormal blood flow or suspected nodules were selected as the imaging section. Sulfur hexafluoride microbubbles (SonoVue, Bracco) were diluted and shaken using 5 mL of saline. Then 2.4 mL was injected via the antecubital vein, followed by a rapid push of 5 mL of saline, and 3-min dynamic ultrasonographic images were monitored and recorded. The CEUS software was used to select the abnormal enhancement range as the region of interest. Specifically, the slice with the clearest abnormal enhancement (mostly hyper-enhanced or heterogeneously enhanced in the arterial phase) was prioritized. Circular regions of interest (ROIs) were manually drawn along the margin of abnormal enhancement in the inner and outer glands, respectively. A total of four ROIs were selected: two on the abnormal enhancement area and two on normal tissue within the same field of view. Areas with calcification, cystic necrosis, gas, capsule, or normal organs were carefully avoided. Then, the time-intensity curve (TIC) of the lesion was analyzed and fitted to derive the contrast parameters: peak intensity (PI), time to peak (TTP), rise time (RT), arrival time (AT), area under the curve (AUC), and gradient (Grad). Subsequently, the patient was subjected to transrectal prostate puncture biopsy. An 18G puncture needle was used to systematically puncture 12 needles at the base, middle, and tip of the inner and outer glands of the right and left lobes of the prostate, respectively, and one or two additional needles in the areas of abnormal contrast enhancement, which were labeled and sent to pathology.

### 2.4 Extraction of exosomes

Exosomes were isolated from serum by differential ultracentrifugation as previously described with minor modifications [4], [5], [6]. Briefly, 10 mL of peripheral venous blood was drawn from the patients, and serum was obtained by high-speed centrifugation and stored in a −80 °C refrigerator. The serum samples were removed from the −80 °C refrigerator, leveled with PBS buffer (Biological Industry, 02-024-1ACS), and centrifuged at 1,500×*g*, 4 °C for 15 min in an ultra-high-speed centrifuge (Beckman, 100-XPN). The supernatant was then collected and centrifuged at 10,000×*g*, 4 °C for 30 min. The supernatant was further removed by centrifugation at 14,000×*g* for 30 min at 4 °C. After this centrifugation, the supernatant was transferred to an ultrahigh-speed centrifuge tube, mixed with 1× PBS buffer, and subject to an ultrahigh-speed centrifuge at 110,000×*g* for 120 min at 4 °C. After centrifugation, the supernatant was discarded, and the pellet was resuspended with 1× PBS buffer, followed by another centrifugation at 110,000×*g*, 4 °C for 60 min. After the final centrifugation, the supernatant was discarded, and the trace liquid at the bottom of the centrifuge tube contained the exosomes.

### 2.5 Nanoparticle tracking analysis assay of exosomes

The nanoparticle tracker (ZetaView, MX 110) was calibrated with polystyrene microspheres (100 nm). The sample cell was washed with deionized water and 1× PBS, respectively. The sample was then taken, diluted again with 1× PBS, and injected for detection.

### 2.6 Statistical analysis

Data were analyzed using SPSS 26.0 statistical software. Normally distributed measurements were expressed as mean ± standard deviation, and comparisons were made by independent samples *t*-test; measurements that did not conform to normality were expressed as P50 (P25, P75), and comparisons between groups were made by the Mann-Whitney *U* test. The diagnostic value of serum exosomes and ultrasonographic parameters for BM of PCa was analyzed using receiver operating characteristic (ROC) curve. The combined model was built using binary logistic regression incorporating serum exosome concentration and ultrasonographic parameters. Spearman correlation analysis was used to explore the correlation between exosome concentration and contrast parameters. The test level was *α* = 0.05, and the difference was considered statistically significant at p ≤ 0.05.

## 3 Results

### 3.1 Clinical data outcome analysis

Serum alkaline phosphatase (ALP) and prostate-specific antigen (PSA) values and Gleason score were higher in the PCa BM group than in the NBM group (p < 0.001). And there was no difference in age, blood calcium value and prostate volume between the two groups (p > 0.05) (Table 1).

**Table 1:** Comparison of clinical data and serum exosome concentrations between the non-bone metastasis (NBM) group and the bone metastasis (BM) group.

Indicators	NBM (*n* = 21)	BM (*n* = 19)	*t/Z*	*P*
Age (year-old)	71.76 ±7.62	72.79 ± 4.91	0.501	0.619
Exosome (×E^10^)	3.00 (2.56, 6.97)	5.47 (4.07, 8.53)	2.506	0.012
PSA (ug/L)	42.51 ± 20.22	87.35 ± 27.63	5.937	<0.001
Gleason	7.00 (7.00, 8.00)	8.00 (8.00, 9.00)	3.648	<0.001
ALP (U/L)	90.00 (82.50, 97.00)	121.00 (104.00, 319.00)	5.080	<0.001
Calcium (mmol/L)	2.24 ± 0.06	2.22 ± 0.07	0.747	0.460
Volumes (×10^4^ mL)	3.80 ± 2.07	5.65 ± 4.24	1.725	0.097

PSA, prostate-specific antigen; ALP, alkaline phosphatase.

### 3.2 Comparison of serum exosome levels between the non-bone metastasis (NBM) and BM groups of PCa

**Figure 1:**
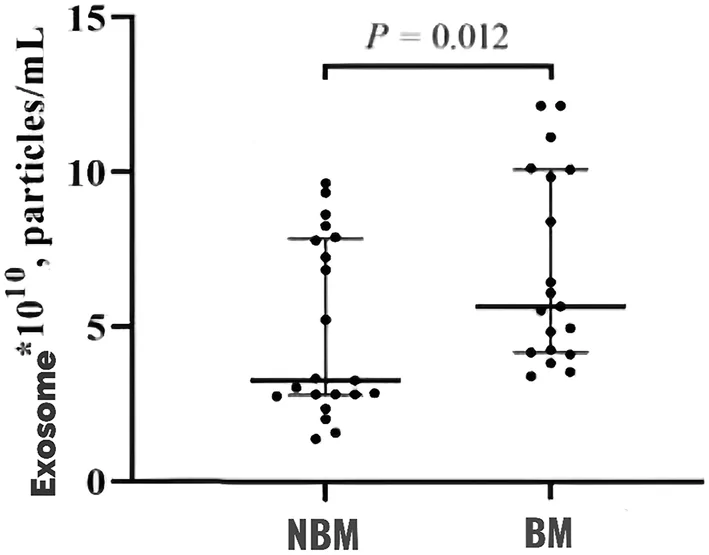
Kruskal-Wallis H test for total serum exosomes in the non-bone metastasis (NBM) and bone metastasis (BM) groups.

The total serum exosome concentration of patients in the BM group of PCa was higher than that of patients in the BM group of PCa, and the difference was statistically significant (p = 0.012) (Figure 1).

### 3.3 Comparison of transrectal ultrasonography parameters between the BM and BM groups of PCa

**Figure 2:**
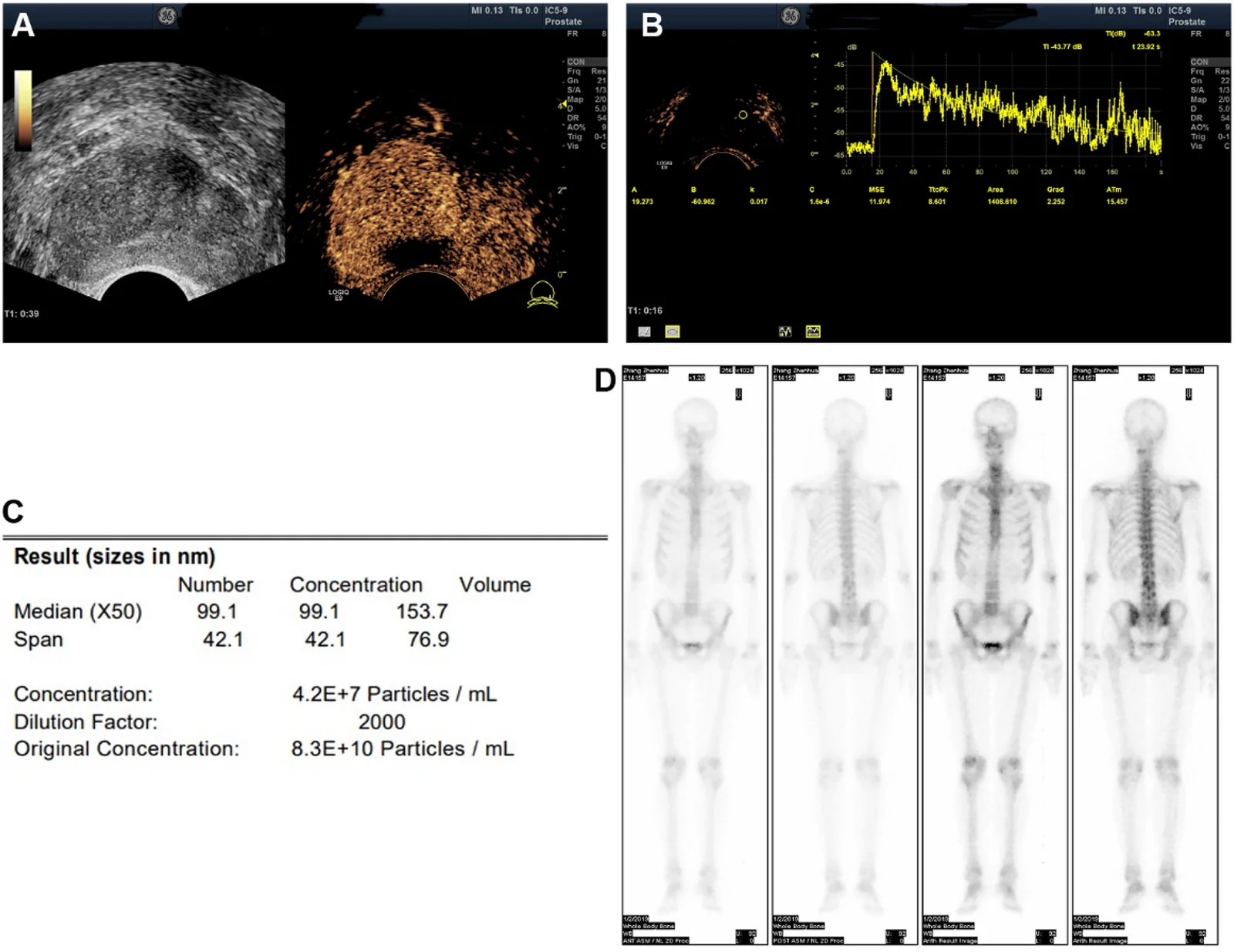
Transrectal ultrasonography, exosome concentration, and whole-body bone scan in a patient with NBM. (A) Prostagram showing non-uniform hyperenhancement. (B) The time-intensity curve (TIC) showed an AUC of 1,041.528 dB s, a PI of 17.81 dB, and a Grad of 2.054 dB/s; (C) serum exosome concentration was 8.3 × E^10^ particles/mL; (D) the bones appeared symmetrical and well defined on the whole-body bone scan, and no abnormal radio-concentrated shadows were seen. AUC, area under the curve; Grad, gradient; PI, peak intensity.

**Figure 3:**
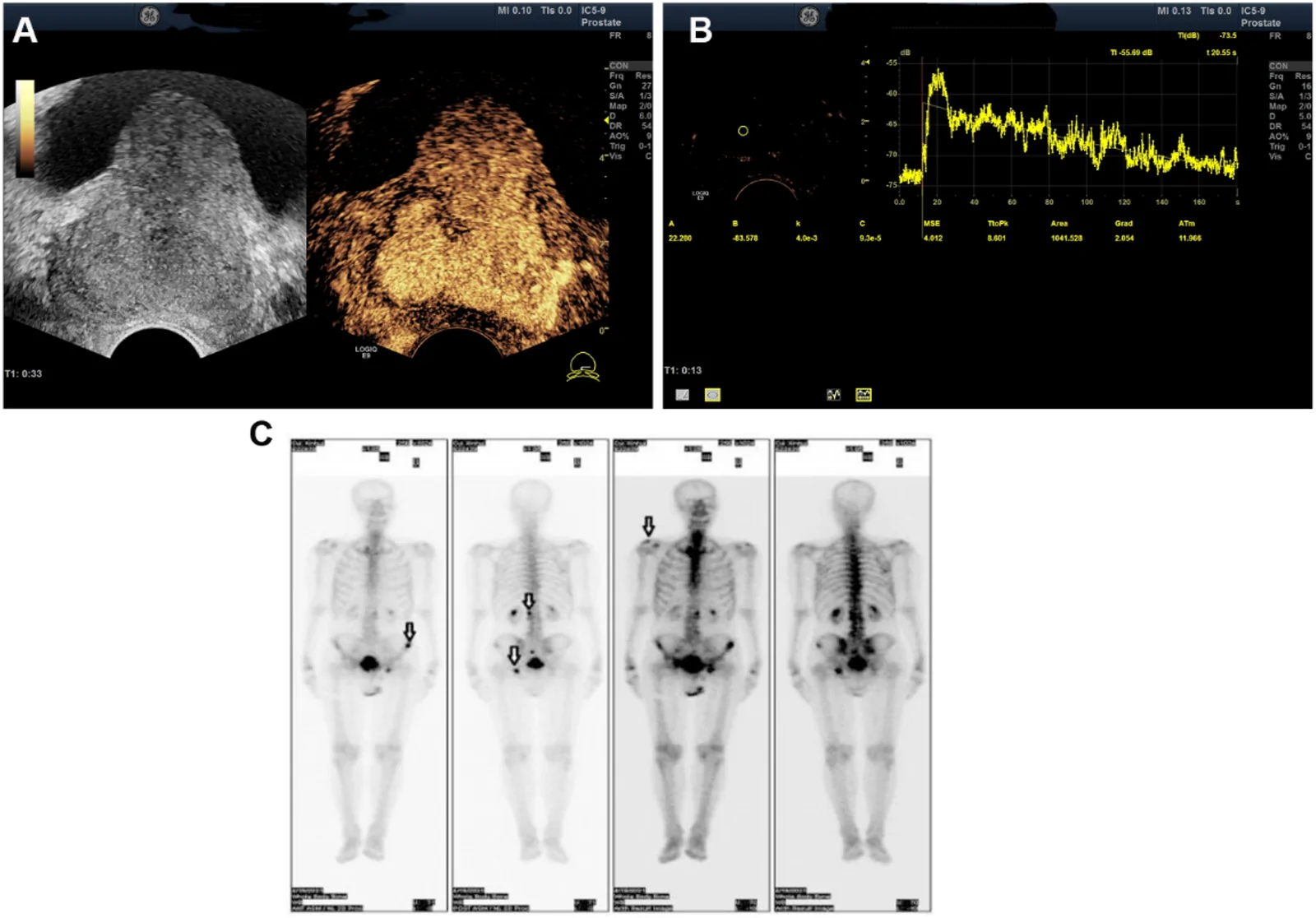
Transrectal ultrasonography, exosome concentrations and whole-body bone scans in patients with BM. (A) Prostagram showing non-uniform hyperenhancement. (B) The TIC curve showed an AUC of 1,408.610 dB s, a PI of 19.53 dB, and a Grad of 2.252 dB/s; (C) whole-body bone scan of a patient with multiple bone metastases involving the second lumbar (L2) and sacral vertebrae.

The values of ultrasonographic parameters PI, AUC and Grad were higher in the PCa BM group than in the PCa BM group (p < 0.05), and the differences in the ultrasonographic parameters AT, RT and TTP were not statistically significant between the two groups of patients (p > 0.05) (Figures 2 and 3, Table 2).

**Table 2:** Comparison of ultrasonographic parameters between the NBM group and the BM group.

Indicators	NBM (*n* = 21)	BM (*n* = 19)	*t*	*P*
RT (s)	10.96 ± 3.55	13.68 ± 5.72	2.059	0.056
AUC (dBs)	1,121.88 ± 58.30	1,187.96 ± 50.93	3.799	0.001
Grad (dB/s)	1.64 ± 0.52	2.13 ± 0.60	2.792	0.008
AT (s)	18.52 ± 3.70	16.93 ± 4.31	1.254	0.218
TTP (s)	29.28 ± 6.39	30.77 ± 8.65	0.623	0.537
PI (dB)	17.80 ± 0.59	19.13 ±0.87	5.679	<0.001

RT, rise time; AUC, area under the curve; Grad, gradient; AT, arrival time; TTP, time to peak; PI, peak intensity.

### 3.4 The value of contrast parameters PI, AUC, Grad and serum exosomes alone and in combination in assessing BM in PCa

**Figure 4:**
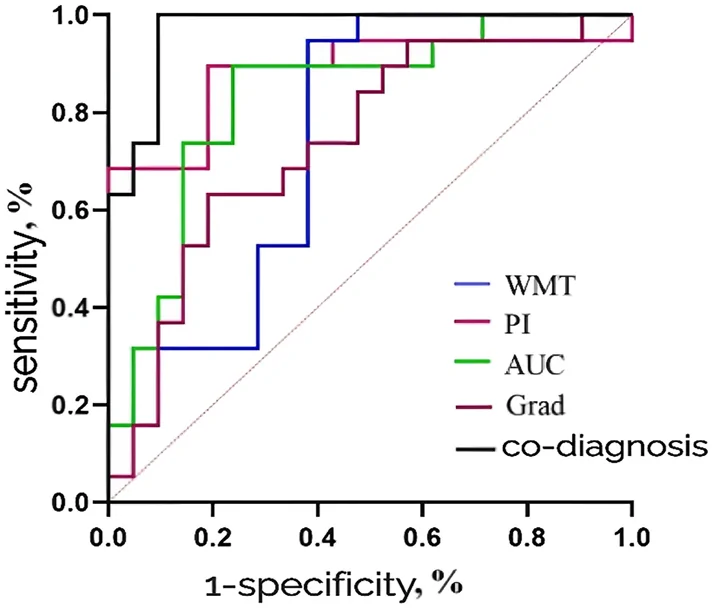
ROC curves of serum exosomes and ultrasonographic parameters alone and in combination for the diagnosis of prostate cancer bone metastases.

According to the ROC curve plot for distinguishing PCa NBM from BM, PI, AUC, Grad and total serum exosomes were shown to be the best cutoff values at 19.33 dB, 1,156.63 dB s, 2.02 dB/s, 3.34 × E^10^ particles/mL, respectively. The AUCs for diagnosing of PCa BM by individual and combined assays were 0.89, 0.83, 0.74, 0.75, and 0.97, respectively (all p < 0.05). The sensitivity was 89.47 %, 89.47 %, 63.16 %, 94.74 %, and 94.74 %, respectively, and the specificity was 80.95 %, 76.19 %, 80.95 %, 61.90 %, and 90.48 %, respectively. The AUC of the combined assay was greater than that of each index alone (Figure 4 and Tables 3).

**Table 3:** Diagnostic value of exosomes and ultrasonographic parameters for prostate cancer bone metastases.

	AUC	Truncation point	Sensitivity (%)	Specificity (%)	Jordon index	*P*
Exosome (×E^10^)	0.75 (0.59, 0.91)	3.34	94.74	61.90	0.57	0.008
AUC	0.83 (0.70, 0.96)	1,156.63	89.47	76.19	0.66	<0.001
PI	0.89 (0.77, 1.00)	19.33	89.47	80.95	0.70	<0.001
Grad	0.74 (0.58, 0.90)	2.02	63.16	80.95	0.44	0.010
Co-	0.97 (0.92, 1.00)	–	94.74	90.48	0.85	<0.001

AUC, area under the curve; Grad, gradient; PI, peak intensity; Co-, combination.

### 3.5 Correlation analysis between ultrasonography parameters and serum exosome concentration

There was no significant correlation between the contrast parameters PI, AUC, Grad, RT, AT, TTP and total serum exosome concentration in the PCa NBM group and the PCa BM group (all p > 0.05) (Table 4).

**Table 4:** Correlation analysis between ultrasonographic parameters and serum exosomes.

Indicators	Exosome			
	NBM		BM	
	*r*_*s*_	*P*	*r*_*s*_	*P*
RT (s)	0.14	0.536	−0.02	0.943
AUC (dBs)	−0.06	0.814	−0.21	0.399
Grad (dB/s)	−0.29	0.201	−0.07	0.770
AT (s)	0.28	0.224	0.38	0.104
TTP (s)	0.19	0.407	0.29	0.231
PI (dB)	0.26	0.249	0.18	0.450

RT, rise time; AUC, area under the curve; Grad, gradient; AT, arrival time; TTP, time to peak; PI, peak intensity.

## 4 Discussion

The rate of advanced BM in PCa is high. Early and efficient identification of BM is clinically important for the follow-up treatment and quality of life of patients.

CEUS allows for the reflection of blood perfusion in tissues, increasing the display and imaging of prostate tumor vasculature [2]. Plotting TIC curves can obtain quantitative contrast parameters and improve the detection rate of PCa. Among the ultrasonographic parameters, PI is the intensity at which the contrast reaches the peak, with higher values indicating that more contrast reaches the lesion; AUC is the perfusion and emptying volume of the contrast medium, with higher values indicating a greater perfusion of contrast medium into the prostate in a given period of time; Grad i.e. the gradient from arrival intensity to peak intensity, with a higher index indicating a faster average perfusion rate of contrast into the lesion. In this study, we found that the values of the contrast parameters PI, AUC and Grad were higher in the BM group of PCa than in the NBM group of PCa (p < 0.05), which is consistent with previous reports in the literature [7]. It indicates that neovascularization in the lesions of BM patients is more than that in the lesions of NBM patients. The tumor growth is closely related to angiogenesis [8]. PCa BM cells proliferate more rapidly, require more oxygen, generate large amounts of neovascularization, and increase microvessel density. At the same time, the tumor blood vessels lack the normal structure of the dendritic distribution, varying in thickness and local dilatation and tortuosity, and are more prone to arteriovenous fistulas and trophoblastic vessels [9].

The occurrence and development of PCa are closely related to the tumor microenvironment. The tumor microenvironment is mainly composed of tumor cells, fibroblasts and various active ingredients produced by them, blood vessels, lymphatic vessels, extracellular matrix, etc. [10]. Exosomes are widely found in many types of cells in the human body and are important carriers of intercellular information transfer. Prostate cell-derived exosomes activate fibroblasts, which affect the tumor microenvironment, inhibit PCa cell apoptosis, promote tumor cell proliferation, and assist in immune escape, thereby promoting PCa progression [11]. In this study, we found that total serum exosome concentrations were higher in patients with bone metastases of PCa than in patients with non-bone metastases. It is suggested that exosomes may have potential application value in the assessment of BM. PCa cells secrete exosomes that can activate neighbouring tumour cells’ microRNAs (miRNAs) or cell receptors, causing tumour cells to continue proliferating. These exosomes can also increase the ability of PCa cells to release more of themselves, resulting in a greater number of them in the patient’s body and further promoting the development and metastasis of PCa [12].

According to the ROC curve plot for distinguishing PCa NBM from BM, the PI, AUC, Grad and total serum exosomes were shown to be the best cutoff values at 19.33 dB, 1,156.63 dB s, 2.02 dB/s and 3.34 × E^10^ particles/mL, respectively. The AUCs for diagnosing BM using individual parameters were 0.89, 0.83, 0.74, and 0.75, respectively, whereas the combined test achieved an AUC of 0.97 (all p < 0.05), with a sensitivity and specificity of 94.74 % and 90.48 %, respectively, indicating significantly superior diagnostic performance over any single indicator. From a clinical applicability perspective, this combined model integrates CEUS, a radiation-free, cost-effective modality that can be seamlessly incorporated into routine prostate biopsy workflows, with serum exosome measurement, a minimally invasive liquid biopsy reflecting systemic metastatic potential [13], 14]. Current clinical strategies for detecting BM in PCa include whole-body bone scintigraphy (BS), PSMA PET/CT, whole-body MRI (WB-MRI), and serum biomarkers such as PSA and alkaline phosphatase [15]. Each has notable limitations. BS, although widely used and inexpensive, suffers from low specificity (approximately 70 %) and is frequently confounded by benign bone conditions such as osteoarthritis or trauma, leading to false positives and unnecessary downstream testing [16]. A recent meta-analysis demonstrated that PSMA PET/CT significantly outperforms BS, with sensitivity of 0.98 vs. 0.85 and specificity of 0.97 vs. 0.70 [16]. However, PSMA PET/CT is expensive, requires specialized radiopharmaceuticals and infrastructure, and exposes patients to substantial ionizing radiation, limiting its use for repeated monitoring or in resource-constrained settings [17]. WB-MRI offers excellent soft-tissue contrast and avoids radiation, but is time-consuming, costly, and contraindicated in patients with claustrophobia or certain metallic implants [15]. Notably, a recent study demonstrated that exosomal miRNAs combined with clinical factors achieved an AUC of 0.956 for diagnosing PCa BM, supporting the value of exosome-based biomarkers [18]. In summary, this exploratory cross-sectional study suggests that the combination of CEUS parameters and serum exosomes may have potential value in the assessment of PCa BM. However, given the study design, these findings are preliminary and warrant validation in larger, multi-center prospective cohorts.

At the same time, this study also found that although ultrasound contrast parameters and total serum exosome concentrations can distinguish between different degrees of PCa, there is no correlation between their concentrations. The possible reasons for this may include the following: (1) Exosomes contain a wide variety of contents, including fusion genes (such as EML4-ALK and ACR-ABL), non-coding RNA (miRNAs, circRNAs, lncRNAs), and proteins. This study detected the total concentration of exosomes in serum samples. (2) Exosomes are closely related to various diseases, such as neurological and immune system disorders [19], 20], so exosomes lack specificity for PCa. (3) Exosomes are present in various body fluids, such as urine, semen, and blood. This study investigated the expression levels of exosomes in serum. The relationship between exosomes in other body fluids and PCa remains to be studied.

There are several limitations of this study. First, the sample size is relatively small (only 40 patients from a single center), and no *a priori* sample size or power calculation was performed. Therefore, the statistical robustness of our findings is limited, and the results should be interpreted as exploratory and preliminary. In addition, the single-center design may limit the generalizability of the conclusions. Larger, multicenter prospective studies with adequate statistical power are needed to validate the application value of transrectal ultrasound contrast imaging combined with serum exosomes in assessing PCa BM. Second, only total serum exosome concentration was measured, without validation of exosome identity (e.g., transmission electron microscopy or exosomal marker proteins) or analysis of exosomal contents. Third, multiple statistical comparisons were performed without correction for multiple comparisons (e.g., Bonferroni correction). As this is an exploratory preliminary study, we did not apply such corrections to avoid overly conservative estimates; however, we acknowledge that this may increase the risk of false positive. Future confirmatory studies should address this issue. Fourth, multivariate analysis was not performed to adjust for clinical confounders (e.g., PSA, ALP, Gleason score) due to the limited sample size. Therefore, whether CEUS parameters and exosome levels are independently associated with BM remains unknown. Fifth, we did not distinguish exosome subtypes (e.g., tumor-derived vs. host-derived). Future studies using tumor-specific markers (such as PSMA or EpCAM) are needed to determine which exosome subset offers better value in the assessment BM.

In conclusion, this exploratory preliminary study suggests that transrectal ultrasound contrast imaging combined with total serum exosomes may have potential value in the assessment of PCa BM. However, due to the limited sample size and single-center design, the findings should be interpreted with caution. Larger, multicenter prospective studies are needed to validate these results and further evaluate the value of this approach in the assessment of BM.
